# *“It’s Opened My Eyes to a Whole New World”:* Positive Behaviour Support Training for Staff and Family Members Supporting Residents With Dementia in Aged Care Settings

**DOI:** 10.1177/15333175241241168

**Published:** 2024-03-27

**Authors:** Alinka C. Fisher, Katrina Reschke, Nijashree Shah, Sau Cheung, Claire O’Connor, Olivier Piguet

**Affiliations:** 1 Disability and Community Inclusion, 64767 College of Nursing and Health Sciences, Flinders University, Bedford Park, SA, Australia; 2 FRONTIER, 90098 Brain and Mind Centre, The University of Sydney, Camperdown, NSW, Australia; 3 7800 University of New South Wales, Sydney, NSW, Australia

**Keywords:** dementia, positive behaviour support, behaviour management, behavioural and psychological symptoms of dementia, challenging behaviours, residential aged care

## Abstract

**Objectives:**

This study examined the acceptability and usefulness of Positive Behaviour Support (PBS) training in enhancing the capabilities of support staff and family members providing behaviour support to residents with dementia in residential aged care (RAC).

**Methods:**

A mixed-methods pilot study was conducted across 3 RAC organisations, involving pre- and post-training questionnaire assessments for clinical leaders (n = 8), support staff (n = 37) and family members (n = 18).

**Results:**

Findings indicated increased confidence among support staff and family members in providing behaviour support, with 96% indicating it would support their practices across settings. Key training benefits included identifying and addressing underlying causes of challenging behaviours. A majority (89%) expressed the need for further behaviour support training.

**Conclusion:**

Recommendations focus on developing systems to enable effective and collaborative behaviour support practices. Further research is needed to examine application of PBS principles and planning for residents living with dementia.

## Significance Statements

1. Participants reported improved knowledge and skills in providing behaviour support to residents with dementia.
2. Further work is needed to build effective and collaborative behaviour support systems in residential aged care.

## Introduction

Dementia is a neurodegenerative disease that affects cognition and everyday functioning, behaviour and emotional regulation.^1^ In Australia, about 188,000 people with dementia live in residential aged care (RAC),^2^ and up to 80%-90% of these residents experience behavioural and psychological symptoms of dementia (BPSD).^3,4^ These include changes such as physical and/or verbal aggression, apathy, disinhibition, loss of insight and compulsive behaviour, which result from a complex interplay between neurological changes, individual characteristics, and environmental factors.^5-7^

Behavioural and psychological symptoms of dementia can negatively impact the quality of life of the person with dementia and their families^8^ and are a key contributor to early transition to RAC.^9^ They also present a significant challenge for service providers,^3,10^ who, when challenged by BPSD, often look to the treating doctor for a ‘quick fix’, often resulting in pressure to prescribe medication.^3^

Pharmacological interventions such as psychotropic medications have shown limited effectiveness in reducing BPSD and are often associated with severe side effects.^11^ Non-pharmacological interventions are therefore recommended as a first-line intervention to address BPSD. Despite this, the recent Aged Care Royal Commission revealed an over-reliance on the use of medication (referred to as ‘chemical restraint’) to manage BPSD, with urgent recommendations for systems reform and person-centred behaviour support practices.^12^ Legislation now directs clinical management away from the use of medications for behaviour management and towards improving quality-of-life,^13^ and specifically requires the development of written behaviour support plans for individual residents in age care.^14^

With the requirement for behaviour support plans to be developed, this raises the question of what behaviour support models are recommended. There are various non-pharmacological approaches shown to benefit people living with dementia, such as the Newcastle Model,^15^ Tailored Activity Program,^16^ Environmental Skill-Building Program,^17^ Reablement,^18^ and the Experiential and Wellbeing Model.^19^ Whilst these approaches focus on meaningful participation and individualised planning, they do not provide specific tools in behaviour analysis and targeted intervention.

In Australia, the CAUSed model has recently been promoted by Dementia Training Australia^20^ as a systematic approach to problem-solving to help support providers minimise the impact of BPSD. This model encourages looking beyond presenting behaviours and to consider why they are occurring (e.g., as a response to environmental factors).^20^ It focuses on modifying ‘triggers’ and meeting the need ‘communicated by the behaviour’. Whilst the CAUSed model places emphasis on person-centred practices that address underlying causes of challenging situations, the scientific basis for analysis and data-informed planning and intervention is unclear. For example, whilst observation (‘ABC recordings’) are recommended, there appears to be no specific model or process recommended for formulation and analysis to inform targeted intervention plans (i.e., those that effectively identify and address causal factors).

Positive behaviour support (PBS) has also recently shown promise as an appropriate non-pharmacological intervention for addressing challenging behaviours across a range of disability types, including brain injury^21,22^ and dementia.^23,24^ Positive behaviour support provides a framework for clinical intervention that has the dual aims of enhancing a person’s quality of life and reducing challenging behaviours. The framework clearly defines a process for assessment and treatment/intervention, can incorporate a variety of evidence-based approaches, and can also be applied in a range of situations or contexts in which challenging behaviours occur. In a dementia context, PBS provides a framework for an individualised process, but one that must flex and adapt to the person’s stage of life and disease progression.^25^ In addition to its promising credentials, PBS is mandated as the required behaviour support framework under the Australian National Disability Insurance Scheme (NDIS), with behaviour support funding under the NDIS available to residents with dementia 65 years or younger.

To improve behaviour support services in aged care, a capable workforce must be equipped to deliver evidence-based behaviour support practices. This requires establishing a unified and standardised set of practice models. Moreover, evidence suggests that staff training programs grounded in a strong theoretical foundation are more successful in reducing BPSD in people with dementia living in RAC.^26^ This aligns with the reported benefits of PBS training, where staff perceived the principles of behaviour analysis, alongside person-centred practice, to offer added benefits compared to other models.^27^ More recently in research conducted by James et al,^28^ the PBS conceptual framework was perceived as helpful by 42 experienced [behaviour support] practitioners in organising their clinical work.

Benefits of PBS staff training have been reported in supporting people living with intellectual disability, including a reduction of challenging behaviours,^29,30^ changed staff behaviour,^31^ as well as positive effects on staff knowledge and perceived confidence.^32^ Further, research suggests interactive PBS workshops with family carers can successfully build capability in providing behaviour support to people living with dementia in home settings, with evidence of practice change (i.e., changes in the way they provided behaviour support), increased confidence and improved relationships.^24^

To date, there have been no PBS training programs trialled in RAC in Australia, nor any behaviour support training programs specifically focused on building collaborative behaviour support practices between support staff and family members. This was the aim of the current study, which sought to build capabilities and confidence of support staff and families in providing behaviour support to people with dementia living in RAC, with a focus on evidence-based and collaborative practice.

## Materials and Methods

### Development of PBS Training Program

Preliminary development of the PBS training program (including content and delivery mode) was informed by research examining PBS training programs and literature in brain injury,^33,34^ dementia,^24,25^ and existing PBS frameworks and practice models.^35-37^ It also built upon recent work examining current practices, experiences and behaviour support needs of family members supporting people living with dementia,^8^ and support staff working in aged care organisations in Australia.^38^

The PBS training program was further informed by a project advisory group, which consisted of 8 members, including family carers, support providers and service managers. This group was established through existing professional contacts in aged care organisations and support from Dementia Australia, Consumer Engagement (in recruiting family carers). The advisory group met 3 times during the development of the program, to inform the design, content, and delivery of the training program. Their input was used strategically in building content where research literature was limited, identifying for example, content relating to collaborative behaviour support practices in aged care settings.

The final training program consisted of 2 phases (see Table 1). Phase 1 comprised intermediate training for clinical/team leaders responsible for developing behaviour support plans/overseeing behaviour support practice (to be trained as ‘PBS Champions’). Phase 2 consisted of introductory training for formal and informal supporters (staff and family members). On completion of the training, the PBS Champions and service manager from each organisation were invited to one follow-up meeting with the PBS trainer. This allowed for reflective discussion regarding PBS principles and content in the context of their current (and unique) systems and behaviour support planning and processes. The intention here, was to support organisations to consider key priorities and areas of focus, with the understanding that organisations may be unfamiliar with PBS principles and processes prior to participating in this program.

**Table 1.** PBS Training Outline.

PBS Champions (Phase 1): Four 3-hour workshops, facilitated online (via Teams)	
1	• Welcome (introduction, project aims, participant involvement)
	• Behaviour changes in dementia and ‘challenging’ behaviours
	• Introduction to PBS: rights-based, person-centred and focused on systems change Introduction to the PBS Pathway model^a^
	• De-escalation strategies; Crisis response
2	• Intro to PBS process (pre-planning, assessment and implementation)
	• Formulation and functional behaviour assessment (FBA); identifying influencing factors to inform intervention (assessment methods, collating and synthesising data)
Homework task (to be completed before session 3): Complete de-identified ABC recordings	
3	• Homework reflections
	• Introduction to preventative and evidence-informed interventions
	• PBS Pathway model^a^ with case examples
	• Basic strategies focused on building capable environments/systems around the person
4	• Monitoring and evaluating PBS plans
	• PBS as a team approach: Collaborative practices between clinical leads, support staff and family members
Support staff and family members (Phase 2): Three 2-hour workshops, facilitated in person	
1	• Welcome (introduction, project aims, participant involvement)
	• Behaviour changes in dementia and defining ‘challenging’ behaviours
	• Behaviour support team and contacts (specific to each organisation)
	• Introduction to PBS: rights-based, person-centred and focused on systems change
	• Introduction to the PBS Pathway model^a^
2	• De-escalation strategies
	• Building capable and meaningful environments reflecting the person’s values and preferences (e.g., positive communication, routine, structure, cognitive strategies, accessibility)
3	• Understanding importance evidence-informed supports (reflecting FBA process conducted by PBS champions)
	• Data collection (why and how); observations and reporting methods
	• The written PBS plan, practice tools, and roles and responsibilities^b^

^a^*An adaptation of the Competing Behaviour Pathway Model,*^40^ *which was used by the trainer to illustrate how elements of PBS process interact with, and inform each other (e.g., how formulation informs targeted behaviour support strategies*. *This model is used on a ‘signpost’ slide to regularly orient participants to PBS process during the training sessions, and as a guide for case examples*.

^b^Including discussion between PBS champions, support staff and family members.

The training sessions with PBS champions (Phase 1) were designed to be delivered online each week over a four-week period. This was to allow PBS champions from different organisations to attend the same sessions, which was not only seen to be an efficient use of resourcing, but also contribute to a richer learning experience for participants. The advisory group also suggested shorter regular sessions (rather than the 2-day workshop initially proposed by the project team), which they felt would be more feasible given time constraints of clinical leaders.

The training sessions with support staff and family members (Phase 2), were designed to be carried out in collaboration with PBS champions at each participating organisation (i.e., in person) across 3 2-hour workshops. This was again considered more feasible (given time constraints and commitments), but at the same time acknowledging the importance of face-to-face sessions in supporting engagement and discussion across the team. Facilitating these sessions at participating organisations was also suggested to promote participation/attendance due to convenience. These were also delivered individually to each organisation as it was important to facilitate confidential (and organisation specific) discussion considerate of specific organisational systems and processes. This design also allowed for flexibility regarding delivery format where required.

All training sessions were facilitated by the same PBS trainer and were guided by PowerPoint presentations. This supported fidelity to the intervention by providing structured content to ensure key components were delivered consistently across participating organisations.^39^ The slides were compiled by the PBS trainer and circulated to the PBS champions for feedback and input prior to the training.

### Terminology

It is important to clarify some terminology used in this PBS education program. There are a variety of terms used to describe behaviours that present challenges for the person living with dementia, family members and the service system. For example, the Dementia Language Guidelines^41^ recommend the use of terms such as ‘changed behaviours’, ‘BPSD’ and ‘expressions of unmet need’. In the current study and PBS education program, however, we use the term ‘challenging behaviour’. This is because PBS is focused on identifying and understanding challenges to find solutions – so articulating challenges in objective terms that help inform targeted formulation and intervention is essential. Consistent with rationale presented in Fisher et al,^25^ the intention is not to label a person as a ‘challenge’, but rather acknowledging that some behaviours present challenges for the person (e.g., negative impacts on relationships and quality-of-life) and the service system (i.e., to address them in a way that is effective and appropriate).

The topic of language and behaviour is of central relevance to this study, with participant training focused on developing shared vocabulary and understanding to support effective practice. To emphasise this point, terms such as BPSD can be problematic in promoting non-pharmacological approaches because they refer to overt behaviours (e.g., verbal aggression) and psychiatric symptoms (e.g., disinhibition, delusion), and as reported in Fisher et al,^25^ each is not best served by one treatment/intervention approach. Indeed, this language in part, may explain the over-reliance on chemical restraints, with behaviour solely viewed as ‘symptoms’ of dementia, which places the onus on dementia in *causing* the challenging behaviour. This may lead to justification for pharmacological ‘treatment’ without acknowledging important causal factors in the environment (and the complex interplay between these factors). Further, ‘changed behaviours’ presents a problem because behaviours will (and do) *change*, but this does not necessarily present a challenge to justify the need/referral for specialist behaviour support (that requires specialist assessment, formulation and intervention).

### Study Design

Pre-test post-test mixed-method design was utilised to pilot the PBS training program with 4 RAC homes (across 3 aged care organisations). Three participant groups/types participated in the pilot, including: (1) allied health or nursing professionals responsible for overseeing/supervising the direct support provided to residents with dementia (to be trained as ‘PBS Champions’), (2) support staff providing direct support to residents with dementia, and (3) family members of residents with dementia being supported by the participating organisation (to complete introductory PBS training). This research was approved by the Flinders University Social and Behavioural Research Ethics Committee (Project number: 5373).

### Participants

#### Aged Care Organisations

Inclusion in this pilot required aged care organisations to be providing residential services to people living with dementia. Potential organisations were identified by advertising via social networks on LinkedIn (social media), the My Aged Care website (https://www.myagedcare.gov.au/) and Dementia Australia (https://www.dementia.org.au/). Organisations who made direct contact in response to the LinkedIn post and organisations listed on these freely available websites were emailed study details, including an introductory letter, and information sheet outlining the details of the study and participant involvement.

The first 4 organisations who registered their interest met inclusion criteria and were invited to participate in the study. The key contact (e.g., service manager) of the organisation was invited to meet with the research team to discuss their involvement, and where organisations consisted of multiple locations/aged care homes, they were asked to nominate one RAC home as the target setting for the pilot.

#### Positive Behaviour Support Champions

Inclusion criteria required that PBS Champions were allied health or nursing professionals, had experience in supporting people with dementia, and at the time of this study, were responsible for overseeing behaviour support practices and direct support provided to residents with dementia within the nominated RAC organisation for the pilot.

The key contact from each participating organisation (e.g., service/clinical manager) was asked to circulate study details to allied health or nursing professionals who met inclusion criteria. This included an information sheet outlining study details and requirements of participation and a consent form. This clearly stipulated that involvement was voluntary, and that a decision not to be involved, would not jeopardize their current role and relationship with their aged care organisation. The first 2 allied health or nursing professionals from each RAC organisation who returned signed consent and met inclusion criteria were invited to participate. There was a total of 8 PBS champions across 4 RAC organisations.

#### Support Staff and Family Members

Support staff and family members were invited to participate in this pilot if they provided direct support (formal or informal) to residents with dementia at the participating RAC homes. These participants were recruited using the same process as for PBS Champions above (i.e., key contact distributing study details and consent from supporters meeting inclusion criteria). The first 20 support staff and 10 families from each organisation who met inclusion criteria and returned signed consent were invited to participate (a total of 80 support staff and 40 family members across the 3 RAC organisations). The recruitment of all participating groups (PBS Champions, support staff and family members) across each participating RAC organisation was confirmed before commencing the pilot to ensure adequate sample sizes.

### Data Collection

In order to gain specific feedback on the content, delivery format and usefulness of the program, this pilot was designed to capture outcome data using questionnaire assessments and semi-structured interviews from the 3 participant groups: (1) PBS Champions, (2) support staff and (3) family members of residents with dementia being supported by the participating organisation. Questionnaire assessments were to be collected via questionnaire assessments conducted prior to training, immediately following the final education session, and at 3 months follow up, including the invitation to participate in a short interview to elaborate on their experiences. However, due to significant missing data from PBS Champions (<50% completion rate) and low response rate from all participants at 3-month follow-up (<20%), despite multiple follow up attempts from the research team, only data from pre- and post-training questionnaires completed by support staff and family members were included for analysis in examining the acceptability and usefulness of the PBS training. However, the demographic information about PBS Champions and participating organisations (collected from PBS champions via the pre-training questionnaire, see below), is summarised in results to provide important context when interpreting results.

#### Pre-Training Questionnaire

#### Positive Behaviour Support Champions

This 25-item questionnaire collected information regarding RAC organisation demographics and current behaviour support practices, included a combination of four-point Likert-type, yes/no, multiple choice and open-ended questions. Ten questions elicited information about the participating RAC (e.g., is it single or multi-site, how many residents have dementia and BPSD) and current behaviour support practices (e.g., who typically writes/develops behaviour support plans, and if behaviour support training is required of support staff). Four questions collected demographic information about PBS Champions (e.g., age, gender, qualification, years of experience, training completed and current role). Four questions gathered information about the residents living with dementia (e.g., primary diagnosis and BPSD). Seven questions asked about current behaviour support practices (e.g., specific model and current approaches), perceived confidence in providing behaviour support (*1 = Not Confident, 2 = Somewhat, 3 = Yes, 4 = Very Confident*), and the level of stress/worry they experience as a result of supporting residents with BPSD (*1 = None, 2 = Some, 3 = Moderate, 4 = Severe*).

#### Support Staff and Family Members

This 11-item questionnaire collected demographic characteristics of supporters and information about current practice. The first open-ended question asked that they describe their ability to manage BPSD (e.g., confidence and specific strategies/approaches they currently use). Three Likert-type questions then elicited information on perceived confidence in providing behaviour support and the level of stress/worry they experience as a result of supporting residents with BPSD. The remaining 7 questions asked about collaborative behaviour support practices (e.g., between clinical leads, support staff and family members) and if they were directly involved in developing behaviour support strategies for residents with dementia they supported.

#### Post-Training Questionnaire

#### Support Staff and Family Members

This 13-item questionnaire elicited feedback about the training model (content, delivery, and format), what aspects of the training they found most/least helpful, and how it could be improved. It also asked about perceived confidence in providing behaviour support since completing the training (e.g., in collecting data and using strategies focused on improving quality-of-life and using strategies across different situations). The final open-ended question asked about what ongoing behaviour support and/or education would be helpful.

### Data Analysis

Quantitative data (Likert-type, Yes/No, multiple choice) responses were imported to a custom designed data extraction table in Microsoft Excel and analysed with descriptive statistics used to report on trends. Four-point Likert-type responses (e.g., ‘Since completing the program, do you feel more confident in providing behaviour support?’ *1 = Not Really; 2 = Somewhat, 3 = Yes, 4 = Definitely*) were combined and reported in 2 categories: ‘Not Really/Somewhat’ and ‘Yes/Definitely’. Supportive open-ended responses, obtained when participants were asked to elaborate or comment on their Likert-type responses, were used to contextualise the quantitative findings. Arguably, this design does not allow us to determine the presence of any significant changes arising from the treatment. By applying relative measures of change, however, this approach enables us to determine change with increased sensitivity, as recommended previously.^24^

Thematic analysis was used to analyse the 6 open ended questions from pre- and post-training questionnaires (e.g., when asked to describe their ability to provide behaviour support [pre-training] and to describe the most helpful aspects of training or ongoing support needs [post-training]). Individual responses to each question were imported to a custom designed data extraction table in Microsoft Excel, where they were coded and then grouped and synthesised into broader conceptual categories according to research objectives. For example, when asked to indicate which elements of the training they felt were ‘most’ helpful, segments of responses *“thinking more about why the behaviour is happening rather than just stopping the behaviour”* and *“learning about the functional based approach”* were grouped within the theme ‘the importance of understanding the behaviour’. Responses to each question were coded and categorised by 2 independent reviewers (authors AF and KR) and were then further refined through reflective discussion between researchers to ensure analysis was representative of the data.^42^ Only participants who completed both pre and post questionnaires were included for analysis. This included 55 datasets (n = 37 support staff; n = 18 family members).

## Results

### Participating Organisations

The pilot was carried out with 3 RAC organisations (O1 = 1 site; O2 = 1 site; O3 = 2 sites). One of the 4 organisations who registered their interest withdrew their involvement before training commenced (due to external circumstances), and consequently, one of the larger participating organisations nominated 2 RAC sites and another nominated 2 homes (wards) within the one site to participate in the pilot. All participating organisations were located in South Australia and were large multi-site aged care organisations consisting of multiple RAC homes and memory support units (MSU) that included residents living with dementia.

The characteristics of each organisation are presented in Table 2. There were between 33% - >70% of residents living with dementia across participating RAC homes, including between 18%-87% with BPSD. Of residents with BPSD, almost all (97%-100%) had a behaviour support plan.

**Table 2.** Participating Organisations - Demographics.

	Residents	BPSD	
	Staff–Resident Ratio	% With Dementia	% With BPSD
O1	1:5-8^a^	33%-64%^a^	18%-87%^a^
O2	1:4.6-6.0^b^	52%	21%
O3	1:6	>70%	50%

^a^Dependant on general accommodation or MSU.

^b^Shift dependant.

### Participant Demographics

#### Positive Behaviour Support Champions

The 8 PBS Champions (2 from each RAC home) consisted of Registered Nurses (RNs) or Clinical Nurses and were primarily female and between 30-55 years of age, with between 7-20 years of experience in supporting people living with dementia. They reported receiving between 0-8 hours of specific behaviour support training prior to their involvement in this program. When asked to indicate what approach/model of behaviour support they were trained in (e.g., CausEd model, PBS), none identified a specific approach (responding with general comments such as *“undergraduate input”* or *“general dementia training”*).

#### Support Staff and Family Members

A total of 37 support staff (S) and 18 family members (F) participated in this pilot across the 3 participating organisations (O1: S = 15, F = 4; O2: S = 12, F = 9; O3: S = 10, F = 5). The 18 Family members participating across organisations were predominately female (O1: 75%; O2: 67%; O3: 60%) and were children (or children-in-law) (67%), with 5 spouses (28%) and 1 sibling (6%).

The demographics of support staff are presented in Table 3. They were predominately female (60%-80%), with over half of support staff across O1 and O3 between the ages of 46-65, and 50% from O2 aged between 18-35. A majority of support staff had either a certificate/diploma or bachelor degree. A majority of O2’s and all of O3’s support staff had received between 0-8 hours of training specific to behaviour support, and a majority of those from O1 had received either ‘no training’ (31%) or ‘17 hours/3 days +’ training (58%) in behaviour support. The main diagnoses of the client group/residents were Alzheimer’s Disease (AD) (71%-100%), Vascular Dementia (VaD) (55%-91%), Mixed type (e.g., VaD and AD together; 55%-91%), Dementia (aetiology not specified; 43%-91%), and FTD (43%-73%).

**Table 3.** Support Staff Demographics.

Organisation	O1 (n = 15)	O2 (n = 12)	O3 (n = 10)
Gender			
Female	12 (80%)	10 (80%)	8 (80%)
Male	3 (20%)	2 (20%)	2 (20%)
Age (years)			
18-25	2 (13%)	3 (25%)	0
26-35	3 (20%)	3 (25%)	0
36-45	1 (7%)	3 (25%)	3 (30%)
46-55	2 (13%)	2 (13%)	4 (40%)
56-65	7 (47%)	1 (7%)	2 (20%)
66+	0	0	1 (10%)
Highest level of education			
Secondary school	2 (13%)	0	0
Certificate/diploma	8 (53%)	1 (12%)	9 (90%)
Bachelor degree	1 (7%)	4 (44%)	1 (10%)
Post graduate degree	4 (27%)	4 (44%)	0
Nil response	0	3	0
Behaviour support training^a^			
No training	4 (31%)	2 (18%)	7 (70%)
1-4 hours	1 (8%)	3 (27%)	2 (20%)
5-8 hours	1 (8%)	3 (27%)	1 (10%)
9-16 hours (1-2 days)	2 (14%)	1 (10%)	0
17 hours + (3 days +)	5 (38%)	2 (18%)	0
Nil response	2	1	0

^a^*Behaviour support training specific to residents with dementia*.

Of the 62% of support staff who received training specific to behaviour support for residents with dementia, none indicated what specific approach/model was used. Of those who provided further detail, this included comments such as *“person centred care”*, *“Dementia Australia training”* and *“general training during Cert III in Aged Care”*.

### Perceived Confidence in Providing Behaviour Support

Prior to completing the training, support staff and family members were asked to indicate their level of confidence in providing behaviour support across 3 domains: (1) understanding why BPSD occur, and (2) identifying and (3) using behaviour support strategies (see Table 4). Approximately half of all support staff indicated they were ‘yes/very confident’ (51%-58%) and almost half indicated they were ‘not confident/somewhat’ confident (42%-49%) across all items. A majority of family members indicated they were not confident across each of these items (73%-82%).

**Table 4.** Support Staff and Family Members Confidence Pre-Training.

	Response	‘Not Confident/Somewhat’ (%)		‘Yes/Very Confident’ (%)	
		S	F	S	F
Understanding why BPSD may occur following dementia	*S = 33* *F = 11*	14 (42%)	8 (73%)	19 (58%)	3 (27%)
Identifying behaviour support strategies	*S = 35* *F = 11*	17 (49%)	9 (82%)	18 (51%)	2 (18%)
Using behaviour support strategies	*S = 34* *F = 11*	15 (44%)	8 (73%)	19 (56%)	3 (27%)

When asked to elaborate on their response, participants who reported being confident in providing behaviour support emphasised their pre-existing experience (e.g., *“many years managing behaviours”* and *“hands on experience”*). For those who indicated being ‘not/somewhat’ confident, comments emphasised continued learning (e.g., *“learning more all the time”* and *“always learning”*).

When support staff and family members were asked how they would describe their ability to manage BPSD, responses were also mixed. Of those who responded (10 support staff and 8 family members), eleven indicated their ability to be good (e.g., responding *“very good”*), including 2 who referred to *“thinking about behaviour”* and *“identifying triggers”* and 4 who reported using positive communication (e.g., *“use a soft tone”*). Four support staff and 6 family members described their ability to provide behaviour support as being poor, for example, responding *“poor”*, to *“learning as I go”*, *“just agree with my sister”* and to *“just do my best”*. The remaining 3 described their ability as *“ok”* or *“basic”*.

### Program Delivery

The PBS training workshops for PBS Champions was facilitated online over 4 weeks (one 2-hr session per week) via Microsoft Teams as designed. For support staff and family members, training was conducted over 3 consecutive days for 1 organisation, and over 3 consecutive weeks (1 session/week) with the remaining 2 RAC organisations to support staff members’ rostering and convenience. Due to a COVID 19 outbreak at one of the RAC homes in O3, training was delayed by a few weeks. Further, the outbreak meant some staff members were no longer able to participate (with their data therefore not included for analysis), and some family members chose to attend training at the other participating RAC home. Subsequently, due to reduced participant numbers and the flexible approach adopted, the 2 data sets (i.e., from both RAC homes) were combined for reporting purposes. A further rationale for this was that the fact that both RAC homes shared a clinical manager and common operational systems.

Following completion of the training workshops as designed, the trainer invited PBS Champions from each organisation to attend a follow-up session (i.e., 1 session for each participating organisation). Positive behaviour support Champions from O1 and O2 attended these sessions, however, no PBS Champions from O3 were available or responsive to the meeting invitation and thus, the clinical manager attended O3’s session in their place. This was considered appropriate to enable the organisation to benefit from this opportunity, and given no specific data was to be collected from this session. Across each RAC organisation these discussions were primarily focused on collaborative practices such as the need to establish communication pathways between service providers (e.g., PBS Champions and direct support staff) and family members, and the need to improve intake processes and early engagement with family to inform effective behaviour support planning. Positive behaviour support Champions and service providers also used this opportunity to discuss processes of data collection (such as A-B-C forms) and analysis. As requested by PBS Champions, case examples using the PBS Pathway model (the ‘PBS mapping tool’ used during the training) were also distributed to all participants (PBS Champions, support staff and family members) for reference. These are available by request from the author.

### Usefulness of PBS Training

#### Most helpful Elements

When asked to comment on what aspects of the training were most helpful in the post-training questionnaire all support staff (n = 37) and family members (n = 18) responded. Three key themes were identified, including (1) ‘the importance of understanding the behaviour’, (2) learning about specific ‘behaviour support strategies’, and (3) the ‘use of case examples’.

##### The importance of understanding the behaviour

Responses emphasised the importance and benefit of understanding why challenging behaviours were occurring and using this knowledge to inform function-based strategies. For example, participants reported: “*Thinking more about why the behaviour is happening rather than just stopping the behaviour” (S21),* learning about *“the functional [function-based] approach” (F4)* and understanding *“possible function…why it is happening” (S49)*. One family member also commented that the training helped in *“identifying early warning signs” (F52)*.

Support staff also emphasised the benefits of learning about data collection processes, including *why* this was important and *how* data is collected through observation. For example, participants reported benefits of: *“ABC [observation data and form]. If in practice in a right way to keep track of why the behaviour occurred and minimise it to reoccur” (S19), “ABC. Possible function – the pay-off for resident displaying behaviour. Why is it happening…” (S22). and “*… *how to use A-B-C properly” (S12)*.

##### Behaviour support strategies

Many participants also reported that practical strategies were helpful. For example, support staff members commented that the training was *“…so good for learning new techniques and different strategies…” (S1),* and that they have *“learnt different techniques to handle different situations” (S30)*. One staff member also reported *“*…*more insight to work more effectively on the floor” (S16)*. One family member commented *“practical tips for dealing with difficult situations” (F16)*.

##### Use of case examples

Support staff and family members reported the benefits of the specific case examples provided to support their learning. For example, participants emphasised the benefits of *“real life examples” (F10)* and *“examples relating directly to my work” (S33)*. One family member also commented that the *“PBS Pathway model made the whole process clear and could see how it all fits together [and] real life examples*…*”* (*F10*).

In addition, some family members reported the training to be helpful in learning more about dementia and common behaviour changes, and the focus on collaborative practice. One participant also reported information on *“how to draw it all together as a team [was] MOST PRACTICAL” (F4)*.

#### Least Helpful Elements

When asked to indicate what parts of the training were least helpful, 30 support staff and family members responded (55% response rate). Of these, 7 (23%) indicated there were no unhelpful elements and instead emphasised the benefits of the training (e.g., commenting *“not a thing”, “all good (very interesting)”*). From the remaining 23 (77%), the following 2 themes were identified: ‘Session length and complexity’ and ‘Combined sessions with staff and family members.

##### Session length and complexity

A few participants reported some sessions to be too long and/or some of the content to be too technical or needing more time to process information. For example, a family member commented that *“it was not unhelpful, but just a bit too quick to take in properly”* (F5). One support staff mentioned that *“the first day was quite technical with a lot of acronyms” (S34)* and suggested *“a glossary of terms would have been useful”*. Another felt the *“the length of the lecture [too long] due to my brain not being used to big words and explanations (I am 77)” (F11)*.

##### Combined sessions with staff and family member

Whilst some staff reported the benefits of having the families present and that it was *“good to have their perspective” (S21)*, others felt that they would have been able to discuss issues more openly if family members were not there. One staff member felt *“...it [training] should definitely be without family members” (S13)* and another reported:

*“Having both staff and family present [meant we were] unable to address specific issues and have honest conversations. Felt uncomfortable & criticised, without family having complete understanding of time constraints and workload.” (S20)*

It is worth noting that these staff members all came from the same organisation (O2). From this organisation, family members indicated that their contributions were not valued, and they were not involved in behaviour support planning. One staff member from O1 commented that *“family member involvement was a great idea in this research program” (S25)*. One family member also felt much of the content was irrelevant to their role, except for the third and final session, which focused more specifically on collaborative practices.

### Impact on Confidence

In the post-training questionnaire, support staff and family members were asked to indicate if the PBS training had an impact on their perceived levels of confidence across 4 domains: (1) their overall ability to provide behaviour support, (2) understanding why BPSD occurs following dementia, (3) identifying appropriate behaviour support strategies, and (4) using behaviour support strategies. Results are presented in Table 5. A majority of support staff (81%-86%) and family members (54%-71%) indicated increased confidence (responding ‘yes/definitely’) across all domains.

**Table 5.** Impact of Training on Supporter Confidence.

	Responses	Not Really/Somewhat		Yes/Definitely	
		S	F	S	F
Ability to provide behaviour support	*S = 36* *F = 5*	5 (14%)	6 (46%)	31 (86%)	7 (54%)
Understanding why BPSD may occur following dementia	*S = 36* *F = 4*	5 (14%)	4 (29%)	31 (86%)	10 (71%)
Identifying behaviour support strategies	*S = 36* *F = 4*	7 (19%)	6 (43%)	29 (81%)	8 (57%)
Using behaviour support strategies	*S = 36* *F = 5*	6 17%	6 (46%)	30 (83%)	7 (54%)

When asked to ‘elaborate’, participants who indicated being more confident (‘yes/definitely’) following the training emphasised improved understanding of the PBS process and data collection. One support staff member who indicated being ‘not really/somewhat’ confident as a result of the training program stated that “Care workers are seen as irrelevant as far as input, we can tell the ENs *[enrolled nurses] any issue that arises, that is as far as it goes for us” (S37)*. A family member also commented that:

*“Some behaviours are difficult to support as a family member. At times I think a change of scenery might help but my parents both require 2 carers and a lifter to get them into a chair and staff levels do not always support this” (F3)*.

#### Transferrable Skills

Support staff and family members were asked if they felt the PBS training would be helpful in supporting them to use behaviour support strategies across different situations with different behaviours. Almost all the supporters (96%, n = 53) who responded to this question indicated ‘yes/definitely’. When asked to elaborate, participants again emphasised the benefit of being prompted to think about and address possible underlying causes of behaviour. Despite reporting benefits, one family member expressed concerns about whether the RAC organisation would value her contribution:

*“I feel empowered to make a difference in improving a person's quality of life. Though I have some reservations about how I would be accepted by the organisation in wanting to become an active contributor…” (F4)*.

Participants were also asked if they felt more confident in identifying the purpose of a behaviour (i.e., its function/reason it occurs) since completing the training. Of the 46 who responded to this item a majority (72%) responded ‘yes/definitely’. When asked to elaborate, support staff and family members emphasised the use of observation methods and collaborative practice between staff and family. Of those who did not feel more confident in this area, comments emphasised the need for more training and support. For example, 1 family member commented that *“these 3 days were an introduction to the potential benefits [of] what I learnt. A refresher, or further updating, would be useful for me to become a contributor” (F4)*. Another family member said she was *“not yet”* confident and would *“need help (from fellow program participants/etc) to put the introductory knowledge into real life practice” (F5)*. One staff member commented that it is *“perfect in theory, but not time to analyse in the real world” (S22)*.

As a result of the PBS training, a majority of support staff and family members (64%; n = 29 of the 45 responses to this question) felt more skilled at collecting data to inform behaviour support planning. For example, a family member stated that the *“data collection component was very helpful” (F3)* and a staff member reported *“A-B-C [observation recording] is now clearer” (S12)*. Some participants who indicated no/limited improvement in data collection again emphasised concerns in translating these skills to practice. For example, one family member said *“the examples were excellent and made sense. Just need collaborative help to do data collection in real life” (F5)*. Another indicated to *“understand the process more, ...would still need more guidance in what/how to observe, keep records and share information” (F10)*.

A large majority of support staff 86% (n = 31) and 54% (n = 7) of family members felt more confident since completing the program (responding ‘yes/definitely’ to this question). Additional comments emphasised the importance of addressing unmet needs and supporting quality-of-life improvements rather than just ‘fixing’ the problem. Those who indicated ‘not really/somewhat’ to this question emphasised their pre-existing skills/focus on supporting quality of life for residents.

### Format/Delivery

A majority of support staff and families (74%; n = 34 of 46 responding participants) found the format of the PBS training program to be appropriate. Specifically, session duration, the amount of information presented and complexity of content (e.g., indicating that this was *“clear and easy to understand” (S22)*), and the mode of delivery. For example, a staff member reported content *“Being broken down into 3 sessions was great - was able to take the time to absorb the content” (S10)*. Another commented that *“2-hours each afternoon was very tiring, but [that the trainers] energy and enthusiasm kept us all paying attention” F16)*.

Nine participants reported that the format of sessions could be improved. One stated a preference for slightly longer sessions (e.g., 2.5-3 hours instead of 2). Another reported that they were *“expecting some individual table discussion”* and that *“this would have enabled better input from family members” (F9)*. One also suggested *“role play”* and the use of *“one real case that actually happens here [in the participating RAC home]” (S13)*, and another suggested more examples using the PBS Pathway model would have been helpful. Others wanted more information on developing written behaviour support plans.

### Ongoing Support Needs and Recommendations

Participants were asked if they would benefit from more ongoing support and/or education about behaviour support. Of the 38 who responded, 89% expressed that more PBS training and practice support was required. These fit within 2 broad themes, including the need for ‘ongoing revision and professional development opportunities’ for staff who completed the training, and the desire for ‘all RAC staff to complete PBS training’. For example, participants reported the desire for an *“annual refresher” (S36)*, with the suggestion of using *“e-learning”* modules *(S24)* and *“online training/course” (S25)* and further *“Role play and or case studies of real-life scenarios” (S7)*.

Support staff expressed the need for *“all staff working in the MSU [to have] more training on PBS” (S16)*. This was also emphasised by family members, who stated that *“all staff at [the aged care organisation] should have the training” (F8)* and *“ALL carers…require the training so that is becomes regular…indeed ESSENTIAL practice” (F4)*. A family member also reported the need for *“more communication with [RAC organisation] leaders”* and to *“educate leaders to be physically more available at the work face – necessary to know both staff and residents…” (F14)*.

## Discussion

This pilot study has provided important insights into acceptability and usefulness of PBS training for staff and families providing support to residents with dementia living in RAC. The majority of participants reported clear benefits, such as increased confidence and improved knowledge and skills in providing behaviour support. There were, however, also significant ongoing support needs reported, and concerns raised about translating this new learning to practice.

The high satisfaction regarding the PBS training program was promising. The most common benefits reported by staff and families were their new understanding of PBS principles and process (e.g., a focus on understanding why behaviours occur to inform preventative strategies), and upskilling in data collection methods (e.g., A-B-C observation charts) to inform evidence-based practices. These findings are consistent with research conducted by Lawson and James^27^ where staff who attended PBS training suggested PBS to be a helpful and consistent framework, and more sophisticated processes beyond simply describing the behaviour/challenging situation.^27^ This finding is also reflected in research conducted by Fisher et al^24^ suggesting PBS education sessions improve the capability of family carers (informal supporters) in providing behaviour support to people with frontotemporal dementia, with key themes relating to ‘recognising the function of behaviours’ and ‘changing their own behaviour’.

For many participants in this study, this was the first time they had received training specific to behaviour support. Indeed, 1 staff member said it opened their eyes a *“a whole new world”* of behaviour support and another reported that they learnt how to collect observation data *“properly”*. These findings reflect the value of having a clearly articulated model/framework for behaviour support planning (e.g., assessment and analysis), which the results of this study suggest may be lacking in RAC. It is perhaps for this reason, that participants appeared to benefit greatly from the PBS Pathway model, which helped to illustrate the relationship between the multiple components of PBS as part of a wholistic process.

Despite the reported benefits of the program, however, some participants appeared to have little confidence that current systems would support new knowledge and skills to translate to practice. This raised concerns that the PBS training may have created false hope for some participants; that they were able to see significant benefits of a PBS approach, but that practice change would not be supported. For some, it is possible this could lead to disengagement (i.e., *“what’s the point*?*”*) or could in fact be an agent of change (i.e., *“now I know what needs to be done*)*”*. As reported by von Treuer et al,^43^ achieving change in the aged care sector is highly challenging, and organisational readiness for change is important to successfully changing workplace practices. This can be enhanced using interventions focused on building practice leadership and work environments prior to implementation of change.^43^

The upskilling of PBS Champions in this pilot certainly hoped to promote practice change, in addition to the collaborative discussion with participants regarding systems barriers and solutions (during training sessions) and the follow-up meetings with organisations to discuss process/planning. Unfortunately, however, the limited engagement from PBS Champions may have fuelled concerns that PBS would not be supported in practice. Future research is needed to identify and assess various factors that can influence PBS implementation, for example using the Consolidated Framework for Implementation Research (CFIR),^44^ with recognition of the challenges of engaging clinical leaders. This may include, for example, competing priorities, time constraints and concerns about the feasibility of PBS, with its limited (but increasing) evidence-base in current literature, in addition to inadequate training and practice support for aged care staff.

The limited training of staff in behaviour support and the disengagement of PBS Champions in the current study highlight the need to clearly define and assign responsibility for developing and overseeing the implementation of behaviour support plans in RAC organisations. This raises the question of whether dedicated staff roles in behaviour support are necessary to ensure effective implementation. Moreover, this requires clarification of the skills required for writing behaviour support plans, and what practice model is recommended. In Australia, The Aged Care Quality and Safety Commission recently released a ‘Behaviour Support Plans’ fact sheet for RAC providers,^45^ which outlines service obligations (e.g., to develop a plan), but does not explicitly address who is responsible (profession, role) or what practice model is to be used. This lack of clarity may, in part explain why staff have received little/no specific training in behaviour support. It may also explain why, in a recent national survey that targeted staff who write behaviour support interventions across aged care and disability sectors in Australia,^46^ that of the 425 respondents, only 1% (n = 6) reported RAC to be their single or primary setting of behaviour support. This raises significant concern, given the legislated requirement for behaviour support plans to be developed, but with very little clarity around who is actually carrying out this work – and who has the skills to do so. More recently, the authors conducted a survey examining experiences of staff providing direct support to people with dementia,^38^ which suggested that behaviour support plans are being commonly developed by RNs, who have very little training in non-pharmacological approaches. However, in research conducted by Ervin et al,^47^ nursing staff reported non-pharmacological interventions to be outside of their domain.

The findings from this study also suggest some staff and family members felt their contributions were not valued or welcomed by service leadership. This is concerning given collaboration with key stakeholders is central to PBS assessment, planning and implementation.^36^ Further, the authors argue that it is the involvement of those providing direct support that keep behaviour support plans relevant and contemporary Fisher et al.^48^ Interestingly, views regarding the involvement of family members in the PBS training also appeared to differ across the organisations. Examining these cultural differences across RAC organisations was not the purpose of this pilot, however, findings emphasise the importance of further investigating the key barriers and enablers of collaborative practice in aged care, which is key to the success of PBS practice.

The importance of regular communication across the support team is further emphasised by dynamic (and rapidly changing) support needs of people living with dementia due to the progressive nature of the disease, which necessitates continually evolving support plans. As reported in Fisher et al,^25^ the PBS framework must be flexible and adaptable to accommodate disease progression. This raises important considerations and concerns regarding the direct translation of comprehensive PBS approaches (e.g., as required under the NDIS)^49^ for people living with dementia later in the progression of their disease (facing end of life), who require more immediate (and best possible solutions) within time constraints. Here, we need specific guidelines/tools to support decision-making around *how* PBS principles might be used to support immediate problem-solving based on best available evidence/information. The argument here, is that in these situations, PBS should not be seen as an ‘all or nothing’ approach, but principles should be considered and applied as needed/deemed best fit.

Of relevance here, is the PBS Pathway model. What was evident during the training, was the benefits of this model in supporting comprehensive planning, but also short-term problem-solving (e.g., to brainstorm potential causal factors and function/purpose of behaviours, and to prompt for function-based planning). This has relevance and warrants further investigation in the context of limited time and flexibility required when supporting people later in progression of their disease - and other situations where comprehensive planning may not be feasible across both aged care and disability sectors.

## Recommendations

The findings of this pilot inform several practice and research recommendations towards building effective behaviour support provision in RAC settings. Whilst PBS training appears to be an acceptable approach to increase the capability of staff in providing PBS to residents living with dementia in RAC homes, practice changes are needed to translate this new learning to practice. For example, it is recommended that service providers:

• Establish clear communication pathways (e.g., regular meetings) between clinical leaders, support staff and family members to support behaviour support planning and practice.
• Provide clarity regarding the roles and responsibilities across behaviour support teams (e.g., who is responsible for training staff, collecting data, developing plans, coordinating meetings and communication with families), considering the unique structures/systems across organisations.
• Ensure/support clinical leaders and support staff involved in behaviour support planning and practice to acquire adequate training (e.g., in PBS/other required models/frameworks), and that families have access to training opportunities and to contribute to behaviour support planning.
• Establish service culture and systems that value and enable collaborative behaviour support practices, which utilise the lived experience of family members from the very beginning (i.e., from intake/transition to a RAC home).
• Provide combined training for support staff and family members to foster effective communication, support knowledge exchange and enable behaviour support planning that leverages collective experience and collaborative practices, but also offer targeted sessions and workshops tailored to the specific roles and responsibilities of the staff and families within the behaviour support team.

Further research is also needed to build effective PBS practice in supporting people living with dementia in RAC homes to ensure predictable and high-quality behaviour support services. This includes examination of:

• The application of PBS principles in the context of dementia to inform effective, immediate, and person-centred supports for residents as the disease progresses.
• PBS training delivered through an organisational readiness for change lens to support practice change, acknowledging and examining unique culture and systems across service providers.
• The key elements of a capable behaviour support workforce in aged care, including required knowledge and skills across management and service roles.
• How to support effective collaborative practice between support staff and family members and build shared understanding regarding PBS process.
• How to best establish and build systems that value and utilise lived expertise of family members.

## Strengths and Limitations

A strength of this study was its mixed methods design, which facilitated insight into trends, in parallel with learning from participant experiences. This multi-faceted methodology is critical in examining new approaches and informing recommendations towards practice improvements. The study also captured data from 3 participant groups, including clinical leaders, direct support staff and family members, allowing for a more holistic consideration of experiences and support needs.

The study also had limitations that should be considered in interpreting results. Firstly, the involvement of organisations solely from South Australia limits the generalisation of findings to other state-based systems. Additionally, there were challenges in collecting participant data post-training and at the three-month follow-up. This was partially attributed to changing support teams, with some staff resigning or changing roles, and fluctuating family situations. However, limited time and competing priorities as reported in the literature 38 may have played a role. Future research should carefully consider these challenges in methodological design.

This research may have been conducted with certain assumptions regarding the pre-existing knowledge of clinical leaders in basic behaviour support principles, given their role and oversight in behaviour support planning in participating RAC homes. However, the demographic questionnaires revealed a significant lack of training among these leaders, which may have impacted their confidence and engagement in understanding the comprehensive nature of the work and precipitated an impression they were ill-equipped to handle it. Future research should carefully consider how to effectively scaffold the required knowledge and skills to support success.

## Conclusion

The finding from this preliminary PBS training pilot suggest that PBS training is a promising approach for enhancing staff and family capabilities in providing behaviour support to residents with dementia in RAC homes. A majority of participants expressed high satisfaction with the training and a notable increase in confidence in their ability to provide behaviour support following their involvement. However, concerns were raised about the adequacy of systems to efficiently translate newly acquired knowledge into practice. Participants also emphasised the need for ongoing training and support in PBS to consolidate learning and establish effective practices. This research contributes to the growing body of literature examining and seeking to improve behaviour support practices in RAC organisations, which is an urgent priority in Australia. The ultimate goal of this research is to enhance the quality-of-life of people living with dementia and ensure the consistent delivery of high-quality behaviour support services across RAC organisations.

## Data Availability

Data sharing not applicable to this article' and omit the last part of the sentence. We did generate and analyse data sets as part of this study, but due to the multiple and complex data sets (as described in the paper) we decided not to make these available.

## Abbreviations

BPSD: Behavioural and psychological symptoms of dementia
NDIS: National Disability Insurance Scheme
PBS: Positive behaviour support
RAC: Residential Aged Care
